# Environmental Impacts on Hardware-Based Link Quality Estimators in Wireless Sensor Networks

**DOI:** 10.3390/s20185327

**Published:** 2020-09-17

**Authors:** Wei Liu, Yu Xia, Daqing Zheng, Jian Xie, Rong Luo, Shunren Hu

**Affiliations:** 1 School of Electrical and Electronic Engineering, Chongqing University of Technology, Chongqing 400054, China; xiayu@2017.cqut.edu.cn (Y.X.); zdq2016@cqut.edu.cn (D.Z.); 51190717102@2019.cqut.edu.cn (J.X.); hsr71@cqut.edu.cn (S.H.); 2 Department of Electronic Engineering, Tsinghua University, Beijing 100084, China; luorong@tsinghua.edu.cn

**Keywords:** link quality estimation, wireless sensor networks, environmental impact, physical layer parameters, received signal strength indicator, signal-to-noise ratio, link quality indicator, communication distance

## Abstract

Hardware-based link quality estimators (LQEs) in wireless sensor networks generally use physical layer parameters to estimate packet reception ratio, which has advantages of high agility and low overhead. However, many existing studies didn’t consider the impacts of environmental changes on the applicability of these estimators. This paper compares the performance of typical hardware-based LQEs in different environments. Meanwhile, aiming at the problematic Signal-to-Noise Ratio (*SNR*) calculation used in existing studies, a more reasonable calculation method is proposed. The results show that it is not accurate to estimate the packet reception rate using the communication distance, and it may be useless when the environment changes. Meanwhile, the fluctuation range of the Received Signal Strength Indicator (*RSSI*) and *SNR* will be affected and that of Link Quality Indicator (LQI) is almost unchanged. The performance of *RSSI* based LQEs may degrade when the environment changes. Fortunately, this degradation is mainly caused by the change of background noise, which could be compensated conveniently. The best environmental adaptability is gained by LQI and *SNR* based LQEs, as they are almost unaffected when the environment changes. Moreover, LQI based LQEs are more accurate than *SNR* based ones in the transitional region. Nevertheless, compared with *SNR*, the fluctuation range of LQI is much larger, which needs a larger smoothing window to converge. In addition, the calculation of LQI is typically vendor-specific. Therefore, the tradeoff between accuracy, agility, and convenience should be considered in practice.

## 1. Introduction

In the past few decades, wireless sensors networks (WSNs) have drawn much attention from academia and industry. WSNs are multi-hop self-organizing networks composed of hundreds and thousands of sensor nodes, which can monitor and collect various information from the deployed area in real-time. They have been successfully used in many fields, such as military surveillance, environmental monitoring, industrial control, and medical care [1]. Link quality estimation is crucial for WSNs due to their self-organizing characteristics as an effective estimation of link quality is the basis of high network performance. The packet reception rate (*PRR*) is the most direct metric for link quality. Although the *PRR* could reflect link quality directly, it always takes a long time to obtain an accurate estimation. Therefore, it is not sensitive to link changes and cannot adapt to the dynamic characteristics of the wireless channel in WSNs [2].

In view of this, finding more agile link quality metrics has become a hot topic in the past years. Several studies have confirmed that certain mapping relationships exist between some link metrics and *PRR*, such as the Received Signal Strength Indicator (*RSSI*), Signal-to-Noise Ratio (*SNR*), and Link Quality Indicator (LQI). These metrics are easier to get than *PRR* itself. Therefore, link quality could be estimated indirectly by constructing mapping models between these metrics and *PRR*, which improves the estimating agility greatly. Such approaches are often called hardware-based Link Quality Estimators (LQEs).

However, the experimental environment in which the mapping model was obtained in most existing studies is relatively single, and the possible impacts of environmental changes are ignored. There are two important questions to be considered: 1. How does the change of environment affect the link metrics? 2. Can the mapping model be obtained in a specific environment be directly used in other environments? To answer these questions, this paper compares the performance of typical hardware-based LQEs in different environments. Then, the impacts of environmental changes on the applicability of these estimators are analyzed quantitatively. The results show that some link metrics will be affected when the environment changes. Depending on the metrics used, the hardware-based LQEs constructed in a specific environment may not be directly applicable to other environments. Consequently, it is necessary to fully consider the impacts of environmental changes in practice.

The contributions of this study are as follows: (1) A comprehensive survey on the experimental environments and modeling methods in existing studies is presented. (2) Impacts of environmental changes on *RSSI*, *SNR*, and LQI are discussed. The results show that the fluctuation range of *RSSI* and *SNR* is more sensitive to environmental changes, while that of LQI is almost unaffected when the environment changes. (3) Aiming at the problematic *SNR* calculation used in existing studies, a more reasonable calculation method is proposed. It is shown that with the proposed method, more accurate *PRR* estimation could be made, especially when *SNR* is low. (4) Impacts of environmental changes on typical hardware-based LQEs are analyzed. The results show that the environmental adaptability of hardware-based LQEs is completely different. LQI based LQEs is the least susceptible to changing environments.

The rest of this paper is organized as follows. In Section 2, related works are given. This is followed by an experimental setup in Section 3. Section 4 summarizes typical hardware-based LQEs which are classified according to the link metrics they used, such as the *RSSI*, *SNR*, and LQI. The impacts of environmental changes on these LQEs are fully analyzed in Section 5. Finally, conclusions are presented and suggestions are made for future works.

## 2. Related Works

Knowing the *PRR* of neighbors could help sensor nodes to select the next-hop more effectively, which will improve network efficiency. To reduce the fluctuation of *PRR*, some studies use an exponentially weighted moving average (EWMA) to smooth *PRR*. Woo et al. [3] proposed WMEWMA, which combines window averaging with EWMA for low pass filtering of *PRR*. Baccour et al. [4] proposed F-LQE (Fuzzy-LQE), which uses fuzzy logic to fuse four link parameters, namely smoothed *PRR* filtered by WMEWMA, link stability factor, link asymmetry level, and averaged *SNR*. As F-LQE is too stable, Rekik et al. [5] and Jayasri et al. [6] adjusted the link parameters involved in fuzzy logic respectively to achieve more agile and accurate estimations. Opt-FLQE (Optimized version of F-LQE) replaces the link stability factor in F-LQE with the smoothed required number of packet retransmissions [5]. ELQET (Enhanced LQE Technique) also uses four link parameters, namely *PRR* obtained by LQI mapping, *SNR* obtained by Kalman filtering, coefficient of variation of *PRR*, and averaged LQI, to characterize link quality [6]. Liu et al. [7] proposed FaLQE, which realizes link adaptation by dynamically adjusting the smoothing factor according to the fluctuation of the link. Although these works have effectively improved the accuracy and stability, the inherent problem of *PRR* is still not resolved: It always needs to take a long time to obtain an accurate *PRR* estimation [8].

The mapping models between the communication distance and *PRR* can be obtained by counting the *PRR*s at different distances. Then, the communication distance could be used as an indirect link quality metric. Zhao et al. [9] classified the wireless link into three regions according to *PRR* values at different distances, which are connected region, transitional region, and disconnected region. Although the range of transitional region is the largest, links within this region are extremely unstable. Srinivasan et al. [10] found that the percentage of links in transitional region ranges from 5% to 60%, and typical characteristic of these links is bursty. Liu et al. [11] established a mapping model between the communication distance and packet loss rate (PLR) by data fitting. Sun et al. [12] proposed a mapping model between the communication distance and *PRR* by combining the theoretical *PRR* model and the log-normal path loss model.

As *RSSI* and LQI can be obtained from the physical layer directly and are related to *PRR* closely, they have been widely used in LQEs. Popular radio transceivers used in WSNs, such as CC2420 [13] and AT86RF230 [14] all provide *RSSI* and LQI measurements. By analyzing the correlations between *RSSI*, LQI, and *PRR*, Bildea et al. pointed out that *RSSI* is not a good discriminator of link categories, while LQI could effectively distinguish good, moderate, and bad links [15]. Jayasri et al. also pointed out that the correlation coefficient of LQI and *PRR* is higher than that of *RSSI* [6]. By studying the relationship between LQI and PLR in outdoor environments, Shu et al. [16] pointed out that there is a definite relationship between LQI and PLR. Luo et al. [17] fitted a mapping model between LQI and *PRR* using the Cubic model. Carles et al. [18] constructed a piecewise linear model of *PRR* as a function of averaged LQI. Gomes et al. [19] pointed out that only using LQI may overestimate the link quality under bad links. Meanwhile, there are also differences in the definition and implementation of LQI in different radio transceivers. Ye et al. [20] constructed a mapping model between *RSSI* and *PRR* based on logistic regression.

In the IEEE 802.15.4 standard, DSSS-OQPSK (Direct Sequence Spread Spectrum Offset-Quadrature Phase Shift Keying) modulation scheme is used in the 2.4 GHz physical layer. Therefore, *PRR* could be computed using the theoretical bit error rate model and *SNR*, which can be calculated by subtracting background noise from *RSSI*. For instance, Sun et al. [21] and Chang et al. [22] respectively use the theoretical model of DSSS-OQPSK for *PRR* estimation. When there are no co-channel interferences, the background noise usually remains stable for a few seconds or even minutes. As a result, changes in *SNR* with time are mainly caused by changes in *RSSI* [23]. On the other hand, some studies obtained the mapping relationships between *SNR* and *PRR* through data fitting. For instance, Senel et al. [8] use a locally available *SNR*-*PRR* curve to estimate the *PRR*. Some studies also use the combination of *SNR* and LQI to estimate the link quality. Qin et al. [24] estimated the link quality by Effective-*SNR*, which is produced by combining *SNR* and LQI with minimal additional overhead. Liu et al. [25] proposed a lightweight multi-parameter fusion estimator, in which weighted Euclidean distance is used to fuse *SNR* and LQI effectively.

Recently, machine learning algorithms began to be employed to optimize the mapping models, to improve the agility and accuracy of LQEs. Liu et al. [26] proposed a machine learning-based scheme 4C, which uses a naive Bayes classifier, artificial neural network, and logistic regression to train historical data of *RSSI*, *SNR*, LQI and *PRR* offline and predicts *PRR* effectively. Liu et al. [27] proposed a real-time link quality prediction model TALENT, which uses stochastic gradient descent online learning algorithm to train logistic regression classifiers using LQI and *PRR* values. Marinca et al. [28] took LQI as input and utilized a prediction game to construct an expert system model for link quality estimation. Fu et al. [29] proposed RADIUS, a thresholding method based on Bayes theory, which uses mean value and variance of *RSSI* to identify the degradation of links, namely, from good links to bad links. Shu et al. [30] proposed a link quality classification model, which fuses two physical layer parameters LQI and *RSSI* and trains the mean values of them by support vector machine. Sun et al. [22] proposed WNN-LQE, which employs a wavelet neural network to predict *SNR* and its variance, and then estimates link quality quantitatively using the theoretical model between *SNR* and *PRR*.

Table 1 summarizes the main features of existing studies. It can be seen that most studies are conducted in a single environment. Although some studies [5,7,9,10,12,20,21,23,25,26,28] considered two or three different environments, they did not explore the impacts of environmental changes on the applicability of hardware-based LQEs. In fact, WSN applications may face a variety of deployment environments. Although existing studies have conducted an in-depth analysis of the relationships between communication distance, *RSSI*, LQI, and *PRR*, their results still cannot answer the two questions about environmental impacts presented in Section 1.

## 3. Experimental Setup

### 3.1. Experimental Environments

Experiments were conducted using TelosB, which is equipped with an IEEE 802.15.4 compliant radio chip CC2420 and an integrated planar inverted F-style antenna printed directly on the circuit board [31]. TelosB has been widely used in WSNs due to its advantages of small size, low power, and ease of use. TelosB uses TinyOS 2.1 and is programmed with NesC language. TinyOS is an open-source operating system developed by Berkeley, which is specially designed for embedded WSNs.

**Figure 1:**
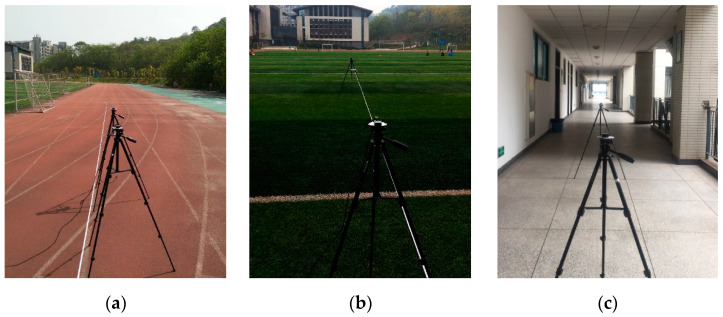
Experimental environments: (**a**) Runway, (**b**) Artificial lawn, (**c**) Corridor.

Several experimental environments were chosen, as shown in Figure 1. Among which, there were not only typical outdoor environments which have simple propagation channels and low external interferences but also a semi-enclosed environment which has complex propagation channel and high external interferences. The corridor was located in the first experimental building of the Chongqing University of Technology. Its length, width, and height were 106, 3.2, and 2.8 m, respectively. It was relatively clean and has almost no obstacles in the corridor. The runway and artificial lawn were located in the playground of the Chongqing University of Technology, which both have no obstacles in the line of sight (LOS). During the experiments, changes in wind speed, temperature, and humidity could be neglected, and there were no other interferences such as walking people.

There were two reasons for choosing the above three environments. First, the propagation characteristics of wireless signals were significantly different in these environments. The surface of the runway was relatively flat. Therefore, the received signal was mainly composed of the LOS component and the reflection component. In addition to the LOS propagation component, there were also scattering components in the artificial lawn, so the composition of the received signal was more complicated. There were many reflective surfaces on the signal propagation path in the corridor, such as the ceiling, ground, and single-sided wall. Therefore, the received signal was a combination of the LOS component and multiple reflection components. These diversities will lead to significant differences in the received signal strength and fluctuation range. Second, the background noise of these three environments were also different. Runway and artificial lawn are typical outdoor environments, in which background noise is typically lower than that in the corridor.

### 3.2. Data Acquiring and Processing

**Figure 2:**
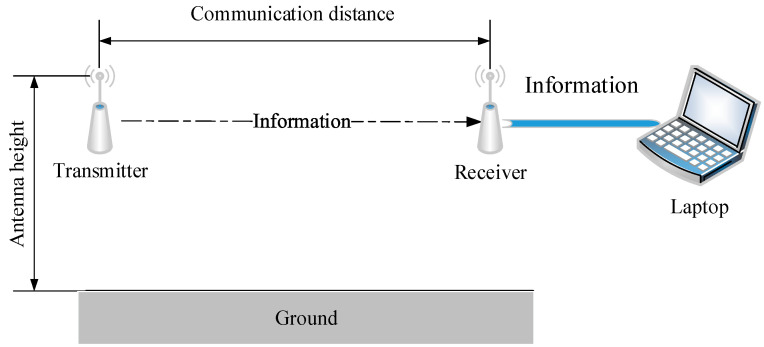
The experiment conducted using two nodes.

Experiments were conducted using two nodes, one as transmitter and the other as receiver. The receiver was connected to a laptop through a serial port, as shown in Figure 2. All experiments were conducted on channel 26 and with 0 dBm transmit power. Antenna height was set to 1.2 m. The communication distance between transmitter and receiver was increased from 0 to 100 m, and the antenna directions of both nodes remained unchanged during this process. Considering that *RSSI* and LQI change greatly when the distance is small, the measurement points were chosen as follows: the step is 0.2 m in the first 5 m, 0.5 m from 5 to 20 m, 1 m from 20 to 60 m, and 2 m from 60 to 100 m respectively. In brief, there is a total of 115 measurement points.

500 packets were sent at each distance, and the inter-packet interval was set to 25 ms. Each packet carried a sequentially increased serial number, so *PRR* could be calculated using the number of successfully received packets. *RSSI*, LQI, background noise power, and serial number of successfully received packets were transmitted to the laptop for analysis, in which *PRR* and the mean values of *RSSI*, *SNR*, and LQI were calculated using MATLAB.

CC2420 provides *RSSI* and LQI measurements, which can be obtained by accessing corresponding internal registers [13]. The received power can be calculated using the *RSSI* value according to the following equation:

(1) $$P=RSSI_{VAL}+RSSI_{OFFSET}$$

where *RSSI_VAL_* is the *RSSI* value provided by CC2420. *RSSI_OFFSET_* is an empirical correction value, which is −45 dBm according to the CC2420 datasheet. LQI presents the quality of received packets, and its values usually range from 50 to 110. The larger the LQI, the higher the quality of the received packet.

## 4. Typical Hardware-Based LQEs: A Survey

**Figure 3:**
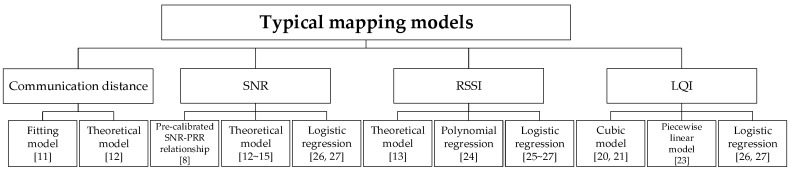
Typical mapping models between indirect metrics and packet reception rate (*PRR*).

According to the analysis in Section 2, typical mapping models for hardware-based LQEs used in existing studies are summarized, as shown in Figure 3. These mapping models are classified according to the link metrics they used, including the *RSSI*, *SNR*, and LQI. For each kind of link metric, the frequently used methods for constructing the mapping model with *PRR* are also given. For example, the theoretical model, polynomial regression, and logistic regression are usually used to construct mapping models between *RSSI* and *PRR* in typical *RSSI* based LQEs. For each kind of modeling method, only one instance was chosen and analyzed in the following chapters.

It should be noted that communication distance is usually used for analyzing and modeling radio links [9,10], and not for online link quality estimation. However, there is already research that utilizes distance to assess the link quality in the design and deployment phase [11,12]. For example, Sun et al. presented a reliability model based on the mapping models between distance and *PRR*, which was used to improve the link estimation and optimize the deployment parameters [12]. Meanwhile, the distance between nodes may be acquired as deployment parameters or measured using appropriate ranging techniques. Therefore, it is meaningful to analyze the influence of environmental changes on these mapping models. With these considerations in mind, these mapping models are also classified and summarized here, although they are exactly not hardware-based LQEs.

### 4.1. Mapping Models between Communication Distance and PRR

According to Figure 3, the theoretical model and fitting model are usually used as mapping models between communication distance and *PRR*. In [12], a theoretical model between communication distance and *PRR* is proposed as follows:

(2) $$PRR={\left(1-Q\left(\sqrt{2\cdot 10^{\left(P_{t}+L_{C}-PL(d_{0})-10n\log_{10}\left(\frac{d}{d_{0}}\right)+X_{\sigma }-P_{n}\right)/10}\cdot B_{N}/R}\right)\right)}^{8l}$$

where *Q*(·) represents the Q function, *P_t_* is the transmit power (dBm), *L_c_* is the signal strength gain (or loss, if its value is negative) in the hardware circuit (dB), *n* is the path loss exponent characterizing the attenuation of wireless signals in the environment, *d* is the distance between the transmitter and receiver (m), *d*_0_ is the reference distance and its value is usually 1 m, *PL*(*d*_0_) is the free-space path loss (dBm) at the reference distance, *X_σ_* is a normally distributed random variable with a mean of zero and a standard deviation of *σ* (dB), *P_n_* is the background noise power (dBm), *B_N_* is the noise bandwidth of the transceiver (kHz), *R* is the communication data rate (kb/s), and *l* is the length of the data packet (bytes). Among which, the value of *PL*(*d*_0_) can be calculated as follows [12]:

(3) $$PL(d_{0})=32.44+20\log_{10}(d_{0}\cdot f)$$

where *f* is the carrier frequency (MHz).

As CC2420 was used in [12], the values of *B_N_* and *R* are 384 kHz and 250 kb/s, respectively. Further, the transmit power *P_t_*, carrier frequency *f*, and packet length *l* were set to 0 dBm, 2480 MHz (corresponding to channel 26), and 17 bytes, respectively. Therefore, substituting Equation (3) into Equation (2), we have

(4) $$PRR={\left(1-Q\left(\sqrt{3\cdot 10^{\left(L_{C}-32.44-20\log_{10}\left(2400\right)-10n\log_{10}\left(d\right)+X_{\sigma }-P_{n}\right)/10}}\right)\right)}^{136}$$

With the measured data in the runway, relevant parameters of Equation (4) were determined, as shown in Table 2. Standard deviation *σ* of the random variable *X_σ_* was calculated from the variances of *RSSI* at different distances, the measured background noise power was used as *P_n_*, and *Lc* and *n* are obtained by the least squares fitting method.

Therefore, the mapping model between the communication distance *d* and *PRR* in the runway could be obtained by substituting the above parameters into Equation (4), as expressed as

(5) $$PRR={\left(1-Q\left(\sqrt{3\cdot 10^{\left(97.6199-20\times \log_{10}\left(2400\right)-15.871\times \log_{10}\left(d\right)+X_{\sigma }\right)/10}}\right)\right)}^{136}$$

In [11], the mapping model between communication distance and *PLR* was constructed by data fitting, expressed as follows

(6) $$PLR=\{\begin{array}{cc} 0, & 0\leq d<d_{1}\\ a_{1}\mathrm{atan}(a_{2}d+a_{3})+a_{4}, & d_{1}\leq d<d_{2}\\ 1, & d>d_{2} \end{array}$$

where *a*_1_, *a*_2_, *a*_3_, *a*_4_ are fitting parameters. With the measured data in the runway, the fitting model between communication distance *d* and *PRR* was obtained, as shown in Equation (7).

(7) $$PRR=\{\begin{array}{cc} 1, & 0\leq d<18\\ 0.5094-0.3401\times \mathrm{atan}(0.3316\times d-13.72), & 18\leq d<82\\ 0, & d>82 \end{array}$$

### 4.2. SNR Based LQEs and Their Mapping Models

According to Figure 3, the theoretical model and logistic regression model (for short, LR model) are usually used as mapping models between *SNR* and *PRR* for *SNR* based LQEs. In [21], the theoretical model was used as the mapping model between averaged *SNR* (defined as *μ_snr_*) and *PRR*, as shown in Equation (8).

(8) $$PRR={\left[1-Q\left(\sqrt{2\times \frac{B_{N}}{R}\times 10^{\frac{\mu_{snr}}{10}}}\right)\right]}^{l}$$

where *Q*(·) represents the Q function, *l* is the number of bits in a packet, *R* is the data rate in kb/s, and *B_N_* is the noise bandwidth of the transceiver in kHz. The values of *R* and *B_N_* are also 250 kb/s and 384 kHz, respectively.

In [26], the mapping model between *μ_snr_* and *PRR* is obtained based on logistic regression. Using the measured data in the runway, a mapping model was obtained, as shown in Equation (9).

(9) $$PRR=\frac{1}{1+e^{3.4435-1.1047\times \mu_{snr}}}$$

### 4.3. RSSI Based LQEs and Their Mapping Models

According to Figure 3, the LR model and polynomial regression model (for short, PR model) are usually used as mapping models between *RSSI* and *PRR* for *RSSI* based LQEs. In [20], the mapping model between averaged *RSSI* (defined as *μ_rssi_*) and *PRR* was obtained based on logistic regression. Using the measured data in the runway, a mapping model was obtained, as shown in Equation (10).

(10) $$PRR=\{\begin{array}{cc} 1, & \mu_{rssi}>-86\\ 1-\frac{1}{1+223.5424\times e^{2.1771\times \mu_{rssi}+198.4593}}, & -96<\mu_{rssi}\leq -86\\ 0, & \mu_{rssi}\leq -96 \end{array}$$

In [19], the mapping model between normalized *RSSI* and *PRR* was obtained based on polynomial regression. As normalized *RSSI* is used, the PR Model is self-adaptive essentially. Therefore, the model given in [19] was used directly:

(11) $$PRR=-3943.5R_{avg}^{6}+6506.6R_{avg}^{5}-4279R_{avg}^{4}+1430.9R_{avg}^{3}-256.47R_{avg}^{2}+23.77R_{avg}^{1}+0.022$$

where *R_avg_* is the mean value of normalized *RSSI* obtained by the median filter, and its value ranges from 0 to 0.5.

### 4.4. LQI Based LQEs and Their Mapping Models

According to Figure 3, the Cubic model, LR model, and piecewise linear model are usually used as mapping models between LQI and *PRR* for LQI based LQEs. In [17], the mapping model between averaged LQI (defined as *μ_lqi_*) and *PRR* is obtained using the Cubic model. Using the measured data in the runway, a mapping model was obtained, as shown in Equation (12).

(12) $$PRR=\{\begin{array}{cc} 1, & \mu_{lqi}>98\\ -0.0000066147\times {\mu_{lqi}}^{3}+0.0010661\times {\mu_{lqi}}^{2}-0.0063\times \mu_{lqi}-2.3975, & 68<\mu_{lqi}\leq 98\\ 0, & \mu_{lqi}\leq 68 \end{array}$$

In [18], the mapping model between *μ_lqi_* and *PRR* is obtained based on the piecewise linear model. Using the measured data in the runway, a mapping model was obtained, as shown in Equation (13).

(13) $$PRR=\{\begin{array}{cc} 1, & \mu_{lqi}>96\\ 0.02492\times \mu_{lqi}-1.392, & 80<\mu_{lqi}\leq 96\\ 0.04986\times \mu_{lqi}-3.389, & \;68<\mu_{lqi}\leq 80\\ 0.00008222\times \mu_{lqi}-0.004111, & \;50\leq \mu_{lqi}\leq 68 \end{array}$$

In [26], the mapping model between *μ_lqi_* and *PRR* is obtained based on logistic regression. Using the measured data in the runway, a mapping model was obtained, as shown in Equation (14).

(14) $$PRR=\frac{1}{1+e^{16.9491-0.2125\times \mu_{lqi}}}$$

## 5. Environmental Impacts on Hardware-Based LQEs

The fluctuation range of *SNR*, *RSSI*, and LQI in different environments was analyzed. Meanwhile, the models between communication distance, *SNR*, *RSSI*, LQI, and *PRR* summarized in Section 4 were also explored in different environments. Root mean squared error (RMSE) of the estimated *PRR* and real *PRR* was chosen as the evaluation index of accuracy, as shown in Equation (15).

(15) $$RMSE=\sqrt{\frac{\sum_{i=1}^{n}{\left(PRR(i)-PRR_{m}(i)\right)}^{2}}{n}}$$

where *n* is the number of samples, *PRR*(*i*) is the practical value of the *i*-th sample, and *PRR_m_*(*i*) is the corresponding estimated value using mapping models.

### 5.1. Environmental Impacts on Mapping Models between Distance and PRR

#### 5.1.1. Communication Distance and PRR in Different Environments

**Figure 4:**
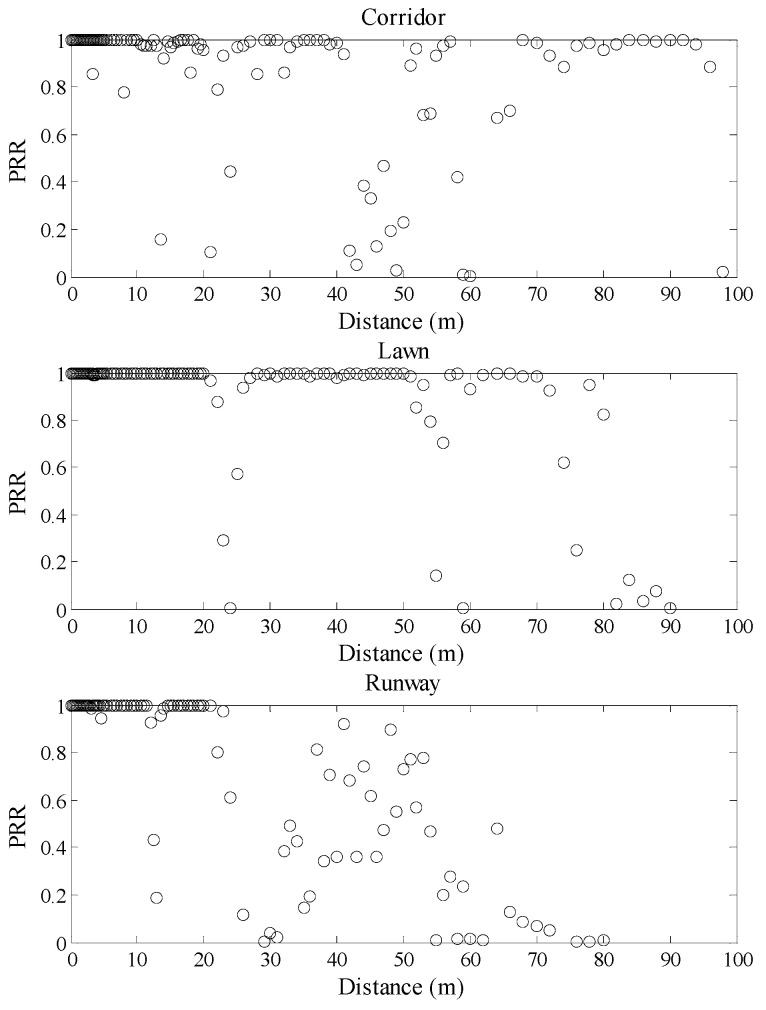
Communication distance vs. *PRR* in different environments.

The relationships between communication distance and *PRR* in different environments are shown in Figure 4. It can be seen that there is no consistent mapping relationship between the communication distance and *PRR* in different environments. The starting and ending distance of the connected region, the transitional region, and the disconnected region are totally different in the three environments. For example, the communication range from 70 m to 90 m belongs to the disconnected region of the runway, while this range belongs to the connected region of the corridor and transitional region of the artificial lawn, respectively. Even the range of the transitional region is also different in the three environments. Moreover, the difference between *PRR* at the same distance is even up to 90%. That is to say, the relationship between communication distance and *PRR* will be greatly affected in changing environments.

#### 5.1.2. Environmental Impacts on Mapping Models between Distance and PRR

**Figure 5:**
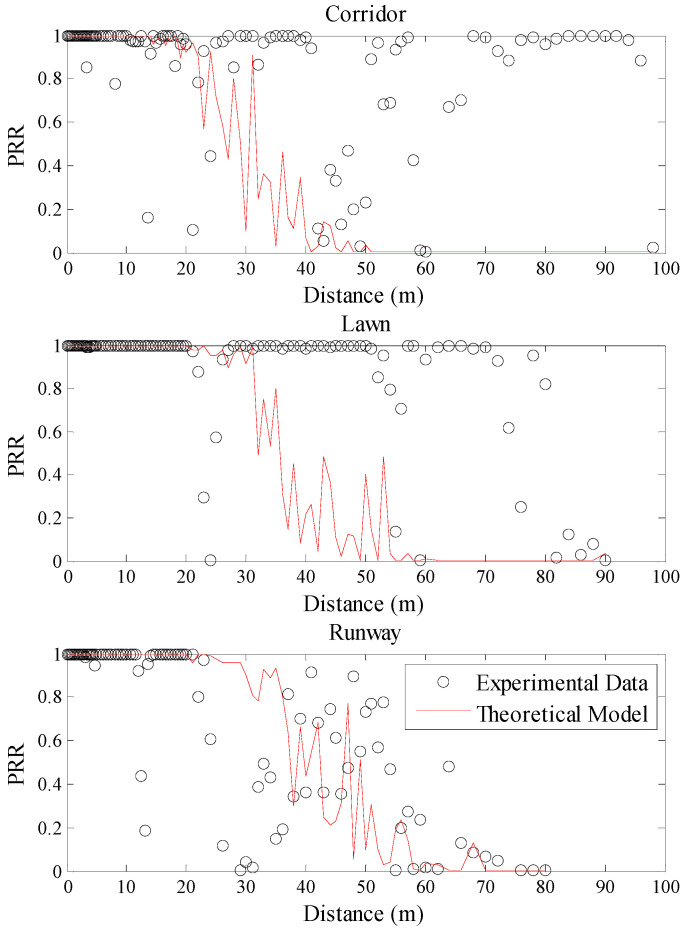
Effects of the theoretical model in different environments.

**Figure 6:**
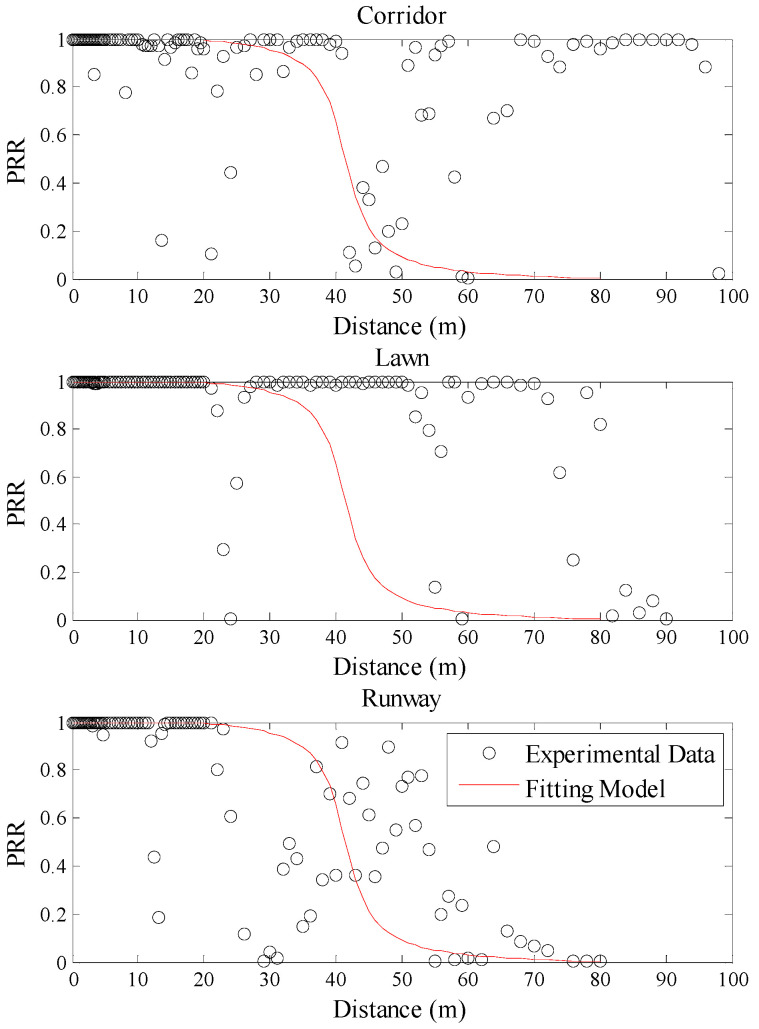
Effects of the fitting model in different environments.

Using Equation (5), the theoretical model between communication distance and *PRR* in three environments was plotted, as shown in Figure 5. As the background noise is easy to be got, the measured noise power of the corresponding environment was used as *P_n_* in the corresponding model. It can be seen from Figure 5 that the theoretical model is basically in agreement with the measured data in the runway. However, it is quite different from the measured data in the other two environments. Even in the artificial lawn which channel condition is closer to the runway, the relationship between communication distance and *PRR* is inconsistent with the theoretical model. Taking a closer look at Figure 5, it can be found that there are some differences between the theoretical curves in three environments. This is caused by the random variable *X_σ_* in Equation (5). Although the theoretical curve obtained for each run will be a little different, the pattern of the theoretical curves is not changed and it does not affect the above conclusions. Using Equation (7), the fitting model between communication distance and *PRR* in three environments was plotted, as shown in Figure 6. From Figure 6, it is obvious that this model is also basically in agreement with the measured data in the runway. It is also quite different from the measured data in the other two environments.

To describe the environmental impacts on these mapping models quantitatively, RMSE of the estimated *PRR* and real *PRR* in the three environments was calculated, as shown in Table 3. It can be seen that even the smallest RMSE is as high as 0.2862. This indicates that it is not accurate to estimate *PRR* using communication distance. On the other hand, RMSEs in the artificial lawn and corridor are much higher than those in the runway when using the mapping model constructed in the runway, no matter whether the model is a theoretical model or a fitting one. Compared with the runway, RMSEs of the theoretical model in the artificial lawn and corridor increase by 57.48% and 85.32%, respectively. Meanwhile, RMSEs of the fitting model in the artificial lawn and corridor increase by 37.95% and 40.31%, respectively. That is to say, the mapping model between communication distance and *PRR* constructed in a specific environment cannot be directly used in other environments.

### 5.2. Environmental Impacts on RSSI Based LQEs

#### 5.2.1. RSSI and PRR in Different Environments

**Figure 7:**
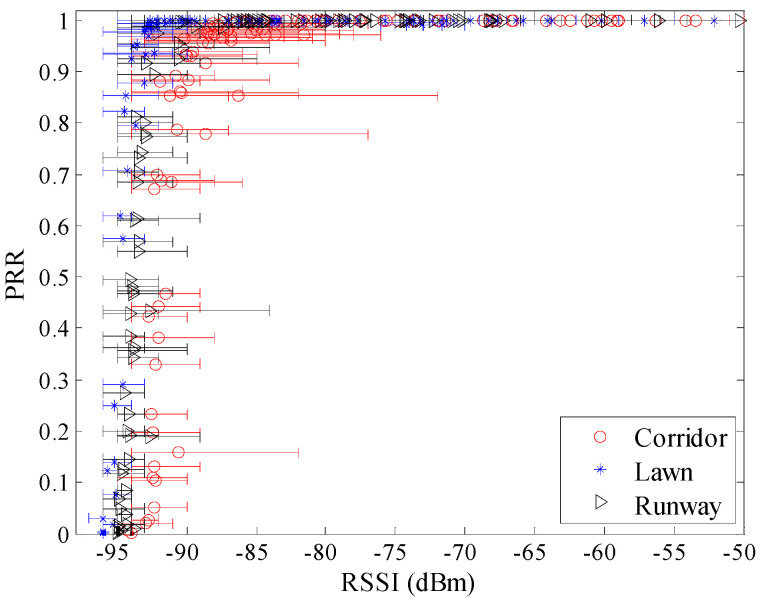
Received Signal Strength Indicator (*RSSI*) vs. *PRR* in different environments.

Figure 7 shows the relationship between *RSSI* and *PRR* in different environments, including the minimum, maximum, and mean value of *RSSI*. It can be seen that the trend of change between *RSSI* and *PRR* is basically the same for the three different environments. In terms of *μ_rssi_*, *PRR* increases as *μ_rssi_* increase: when *μ_rssi_* is lower than −95 dBm, *PRR* approaches 0; when *μ_rssi_* is higher than −90 dBm, *PRR* approaches 100%; when *μ_rssi_* is located between −95 dBm and −90 dBm, *PRR* rapidly increases from 0 to 100%. In terms of the fluctuations of *RSSI*, it is significantly greater in the corridor than that in the runway and artificial lawn.

**Figure 8:**
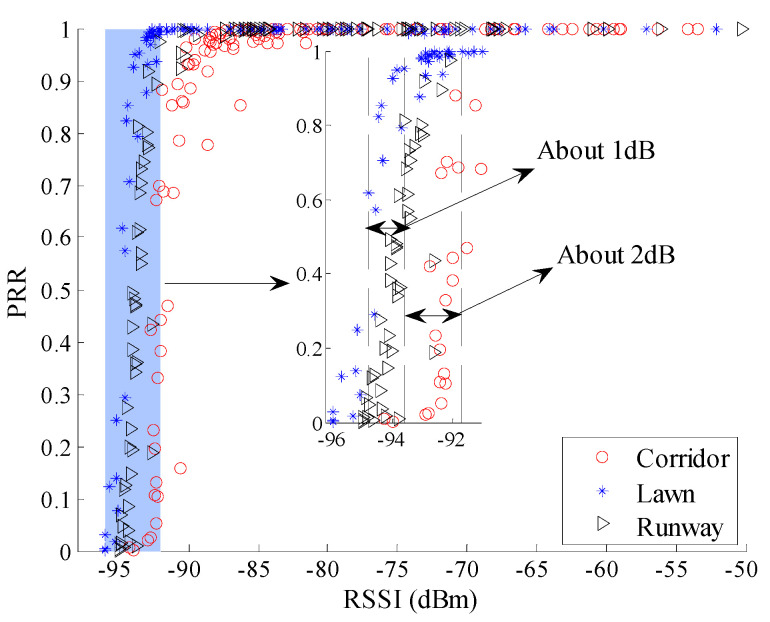
*μ_rssi_* vs. *PRR* in different environments.

To observe the environmental impact on the relationship between *RSSI* and *PRR* more clearly, the relationship between *μ_rssi_* and *PRR* is shown in Figure 8. There already exist some studies which utilize the relationship between *RSSI* and *PRR* to estimate link quality [12,19,20,26,27]. Although the pattern between *μ_rssi_* and *PRR* is basically the same in different environments, it is not difficult to find out that there are still some differences among the relationships between *μ_rssi_* and *PRR* in different environments. For example, compared with the relationship between *μ_rssi_* and *PRR* in the runway, the relationship in the corridor translates to the right by about 2 dB. This means that the received signal power in the corridor should be 2 dB higher than that in the runway to get the same *PRR*. The 2 dB difference may cause misjudgment of the link quality. For example, when *μ_rssi_* is −93 dBm, *PRR* in the corridor is less than 10% which means a bad link, while *PRR* in the runway is greater than 90% which means a good link.

Theoretically, the translation of the relationships between *μ_rssi_* and *PRR* should be caused by the difference in background noise. To confirm this conjecture, background noise in these different environments was measured. The noise power in the corridor, artificial lawn, and runway are −96.20 dBm, −99.61 dBm, and −98.37 dBm, respectively. The difference between the corridor and the runway happens to be 2.17 dB. This indicates that translation of the relationships between *μ_rssi_* and *PRR* in different environments is indeed caused by the difference in background noise.

**Figure 9:**
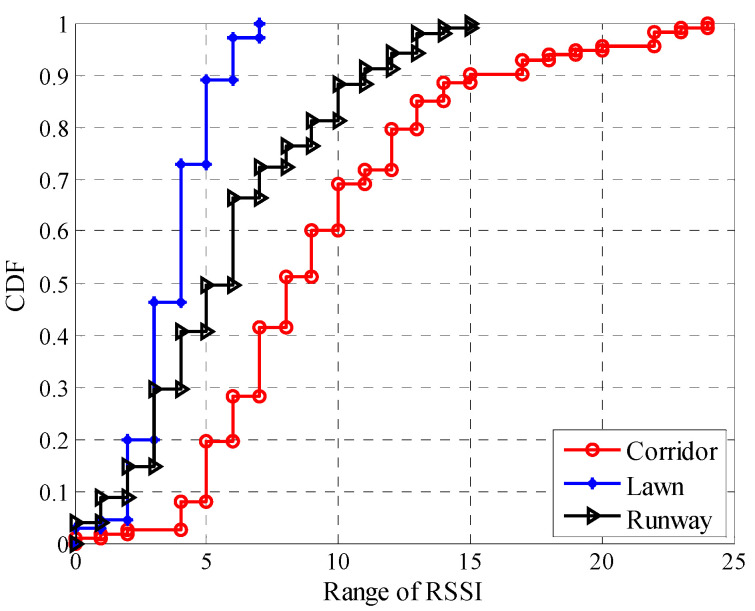
Cumulative distribution functions (CDFs) of the fluctuation range of *RSSI* in different environments.

To describe the impact of environmental changes on the fluctuation of *RSSI* more intuitively, the fluctuation ranges of *RSSI* in different environments were statistically obtained, and their cumulative distribution functions (CDFs) are shown in Figure 9. The fluctuation range of *RSSI* is calculated by subtracting the minimum *RSSI* from the maximum one. It can be seen that the fluctuation range of *RSSI* in the corridor is the largest, with about 30% of the fluctuation range higher than 10 dBm, and about 9.73% of the fluctuation range higher than 15 dBm. In contrast, the fluctuation range of *RSSI* in the artificial lawn is much smaller, with only about 10.91% of the fluctuation range higher than 5 dBm, and 100% of the fluctuation range lower than 10 dBm. The fluctuation range of *RSSI* in the runway is between that in the corridor and artificial lawn. The fluctuation range of *RSSI* is mainly determined by the number of propagation paths. The more the factors that cause signal reflection, diffraction, and scattering, the greater the fluctuation range of *RSSI*.

#### 5.2.2. Environmental Impacts on RSSI Based LQEs

**Figure 10:**
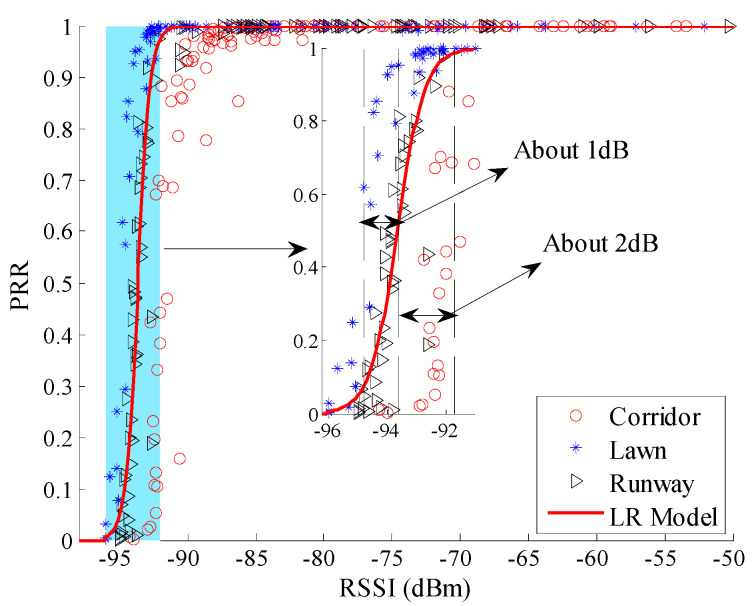
Effects of the logistic regression (LR) model in different environments.

**Figure 11:**
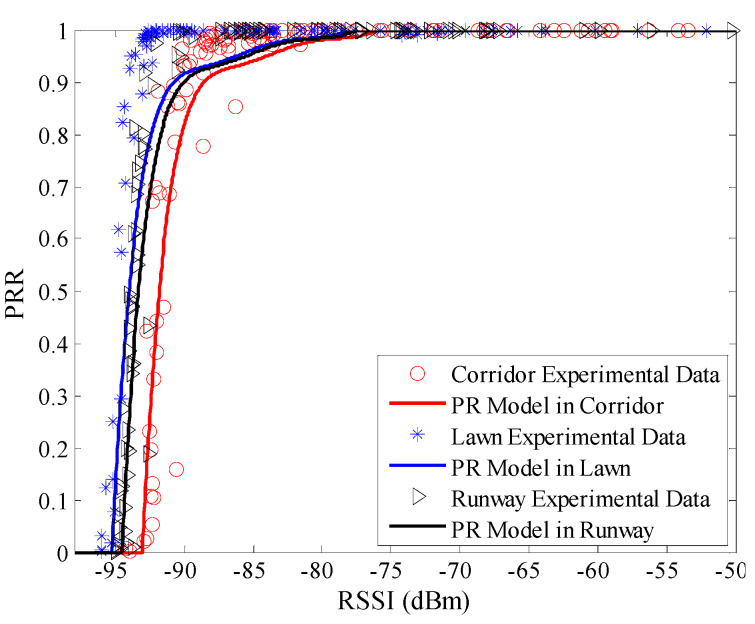
Effects of PR (polynomial regression) model in different environments.

Using Equation (10), the LR model between *μ_rssi_* and *PRR* in three environments was plotted, as shown in Figure 10. It is clear that only the measured data in the runway are nearly coincident with the LR model. There are obvious translations from the model curve to the measured data in the artificial lawn and corridor. Using Equation (11), the PR model between *μ_rssi_* and *PRR* in three environments was plotted, as shown in Figure 11. Unlike the LR model, there is no obvious translation from the PR model to the measured data in the artificial lawn and corridor.

To describe the environmental impact on *RSSI* based LQEs quantitatively, RMSE of the estimated *PRR* and real *PRR* in three environments were calculated, as shown in Table 4. It can be seen that RMSEs of the LR model in the artificial lawn and corridor are much higher than that in the runway. Compared with the runway, RMSEs of the LR model in the artificial lawn and corridor increase by 40.74% and 131.96%, respectively. It can be concluded that the LR model is not suitable for both the artificial lawn and corridor. That is to say, the LR model between *μ_rssi_* and *PRR* constructed in a specific environment cannot be directly used in other environments.

On the other hand, the RMSEs of the PR model in the three environments are quite close. Compared with the runway, RMSE of the PR model in the artificial lawn increases by 13.32%, but RMSE in the corridor reduces by 12.07%. Thanks to the normalization of *RSSI*, the PR model is adaptive to the environment to a certain extent. However, it is obvious from Figure 11 that the PR model is not in good agreement with the measured data when *PRR* changes from 0.8 to 1.0. The environment in which the PR model was constructed is a typical industrial environment [26], where high interference may exist. This is also explained from the side why the RMSE in the corridor is the smallest, as the corridor is the closest to the industrial environment.

### 5.3. Environmental Impacts on SNR Based LQEs

#### 5.3.1. SNR and PRR in Different Environments

**Figure 12:**
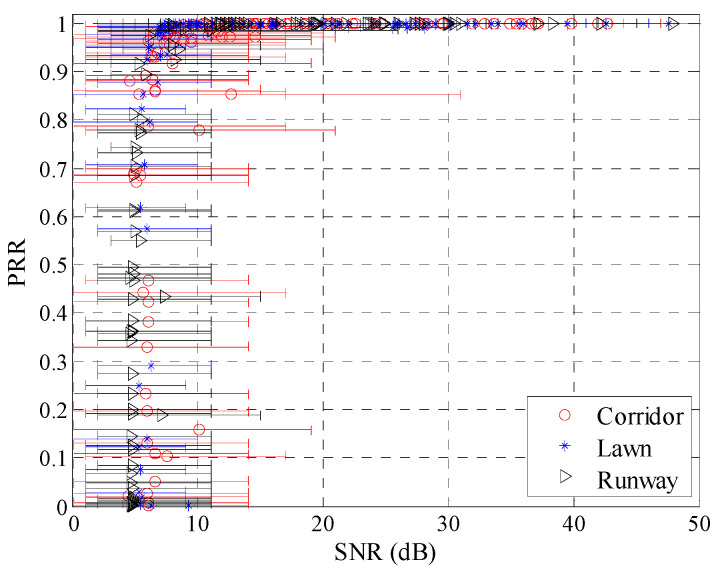
*SNR* vs. *PRR* in different environments.

Figure 12 shows the relationship between *SNR* and *PRR* in different environments, including the minimum, maximum, and mean value of *SNR*. Generally, *SNR* is calculated as follows [12,21,22]:

(16) SNR=RSSI−N

where *RSSI* and *N* are the received signal power and background noise power in dBm. It can be seen that the trend of change between *SNR* and *PRR* is basically the same for the three different environments. In terms of *μ_snr_*, *PRR* increases as *μ_snr_* increases. When *μ_snr_* is lower than 4.5 dB, *PRR* approaches 0; when *μ_snr_* is higher than 8 dB, *PRR* approaches 100%; when *μ_snr_* is located between 4.5 dB and 8 dB, *PRR* rapidly increases from 0 to 100%. In terms of the fluctuations of *SNR*, it is significantly greater in the corridor than that in the runway and artificial lawn.

**Figure 13:**
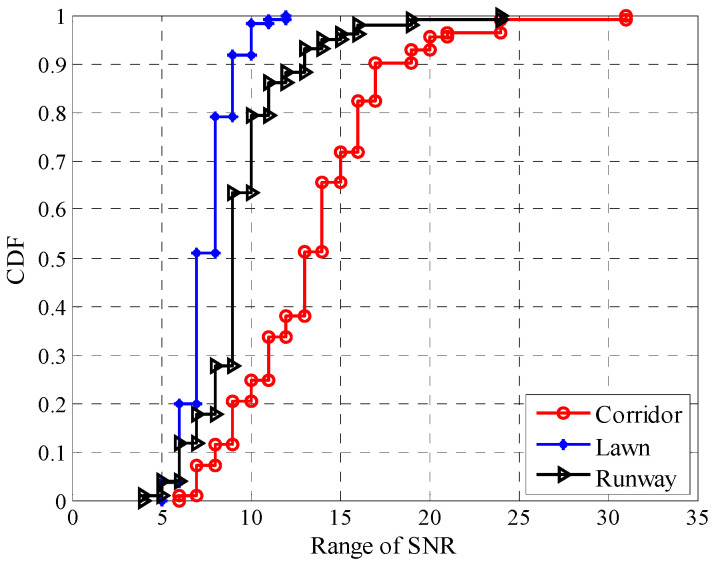
Cumulative distribution functions (CDFs) of the fluctuation range of *SNR* in different environments.

To describe the impact of environmental changes on the fluctuations of *SNR* more intuitively, the fluctuation ranges of *SNR* in different environments were statistically obtained, and their CDFs are shown in Figure 13. The fluctuation range of *SNR* is calculated by subtracting the minimum *SNR* from the maximum one. It can be seen that the fluctuation range of *SNR* in the corridor is the largest, with more than 80% of the fluctuation range higher than 10 dB, and about 30% higher than 15 dB. In contrast, the fluctuation range in the artificial lawn is much smaller. There is less than 2% of the fluctuation range higher than 10 dB, while none is higher than 15 dB. The fluctuation range in the runway is between the corridor and artificial lawn, with about 20% of the fluctuation range higher than 10 dB and about 3% higher than 15 dB.

**Figure 14:**
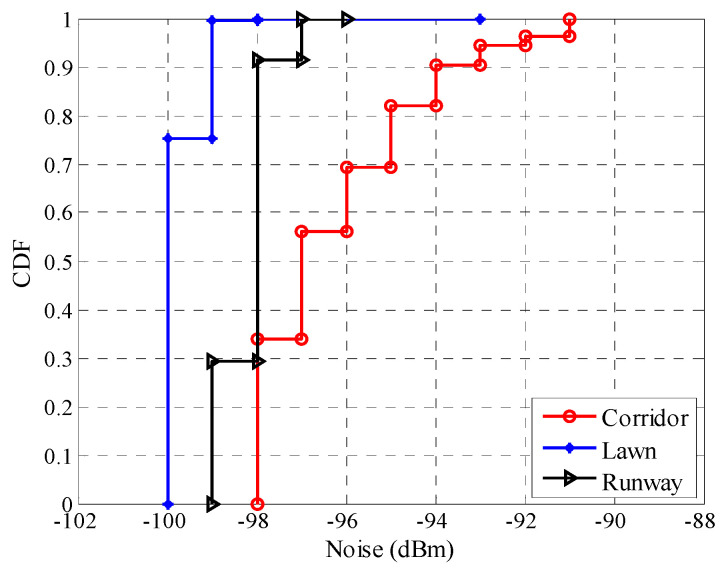
CDFs of the background noise in different environments.

Furthermore, the background noise in these different environments was also measured, and their CDFs in different environments are shown in Figure 14. It is clear that the fluctuation range of background noise in the corridor is the largest, and that in the lawn is the smallest. From Equation (16), it is obvious that *SNR* is related to both *RSSI* and background noise. Combined with Figure 9 and Figure 13, it is reasonable to say that background noise is also contributed to the fluctuation range of *SNR* in different environments.

**Figure 15:**
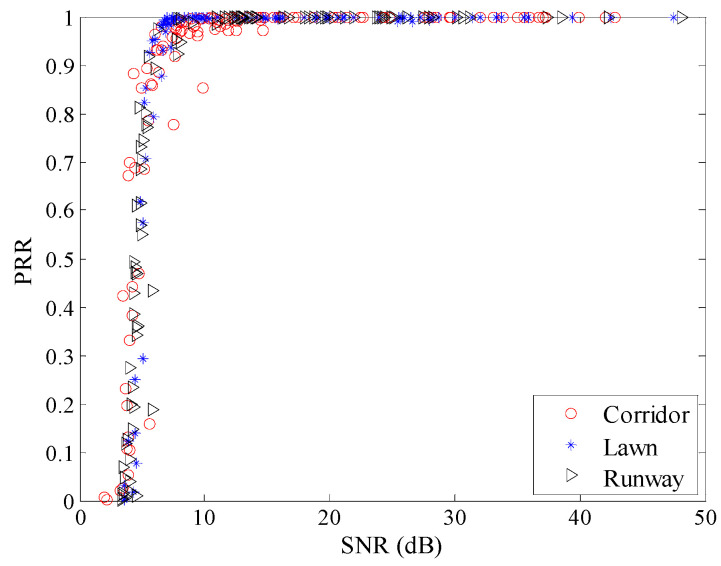
*μ_snr_* vs. *PRR* in different environments.

To observe the environmental impact on the relationship between *SNR* and *PRR* more clearly, the relationship between *μ_snr_* and *PRR* is shown in Figure 15. There already exist some studies which utilize the relationship between *SNR* and *PRR* to estimate link quality [8,21,22,23,24,26,27]. It can be seen that there is no obvious difference between the relationships between *μ_snr_* and *PRR* in different environments. This means that the relationship between *μ_snr_* and *PRR* is almost unaffected when the environment changes.

#### 5.3.2. Environmental Impacts on SNR Based LQEs

**Figure 16:**
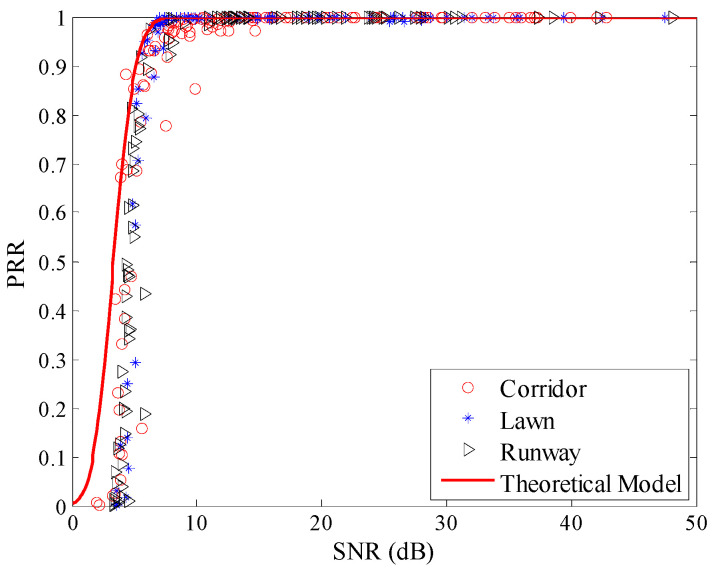
Effects of the theoretical model in different environments.

Using Equation (8), the theoretical model between *μ_snr_* and *PRR* was plotted, as shown in Figure 16. The measured data in all three environments are not coincident with the theoretical model. There is an obvious deviation from the model to the measured data. To describe the environmental impact on the theoretical model between *SNR* and *PRR* quantitatively, RMSE of the estimated *PRR* and real *PRR* in the three environments were calculated, as shown in the first row of Table 5. It can be seen that RMSEs in all three environments are quite large.

It looks like that the theoretical model between *SNR* and *PRR* is invalid. However, when getting down to the details, we can find that the calculation of *SNR* in Equation (16) is problematic. *SNR* is calculated by subtracting the measured noise power (*N* in dBm) directly from the measured signal power (*RSSI* in dBm). However, the transceiver only measures the power at the antenna without attempting to distinguish whether it is due to signal or noise. That is to say, the noise power also contributes to the *RSSI* value. Therefore, the actual ratio of the signal power to noise power should be:

(17) $$\gamma =\frac{P_{RSSI}-P_{N}}{P_{N}}=\frac{P_{RSSI}}{P_{N}}-1=10^{\frac{RSSI-N}{10}}-1$$

where *P_RSSI_* and *P_N_* are corresponding values in mW for *RSSI* and *N*, respectively. Consequently, the *SNR* in dB should be expressed as follows

(18) $$SNR=10\log_{10}\left(10^{\frac{RSSI-N}{10}}-1\right)$$

**Figure 17:**
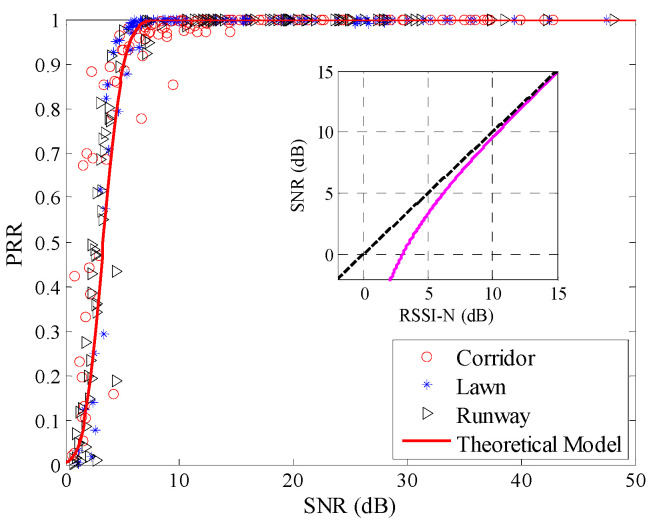
Effects of the theoretical model after correction.

Figure 17 shows the theoretical model after correction. It is obvious that after correction, the theoretical model coincides well with the measured data. The relationship between *SNR* and *RSSI*-*N* is also shown in Figure 17. It can be seen that the actual *SNR* is nonlinear with *RSSI*-*N*, especially when *RSSI*-*N* is small. That explains why the original theoretical model deviates from the measured data, especially when *SNR* is small, as shown in Figure 16.

**Figure 18:**
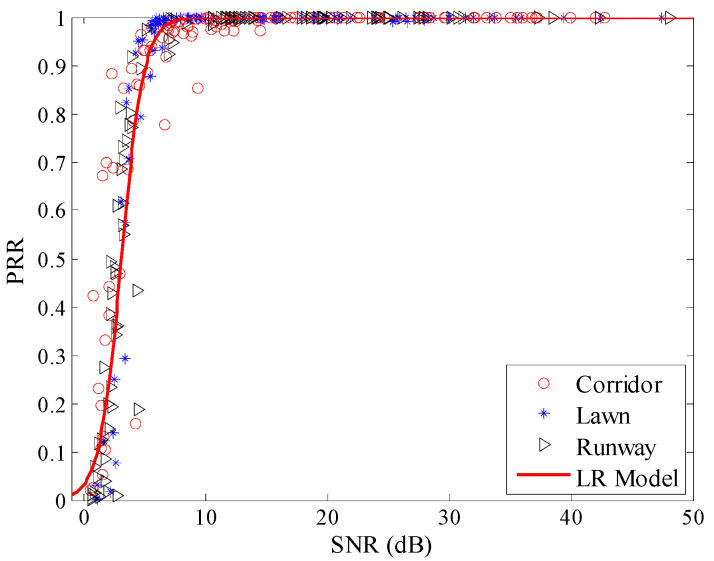
Effects of the LR model in different environments.

Using Equation (9), the LR model between *SNR* and *PRR* was plotted, as shown in Figure 18. From Figure 18, it is obvious that estimated values gained by the LR model basically coincide with the measured data.

RMSEs of the estimated *PRR* and real *PRR* in three environments were also calculated for the theoretical model after correction and the LR model, as shown in Table 4. After correction, RMSEs of the theoretical model in all three environments are reduced effectively, which means that the proposed calculation method of *SNR* is reasonable. Compared with the runway, RMSE of the theoretical model in the artificial lawn reduces by 51.40%, but RMSE in the corridor increases by 15.08%. Compared with the runway, RMSE of the LR model in the artificial lawn reduces by 42.89%, but RMSE in the corridor increases by 11.27%. That is to say, the *SNR* based LQEs are almost unaffected by environmental changes.

### 5.4. Environmental Impacts on LQI Based LQEs

#### 5.4.1. LQI and PRR in Different Environments

**Figure 19:**
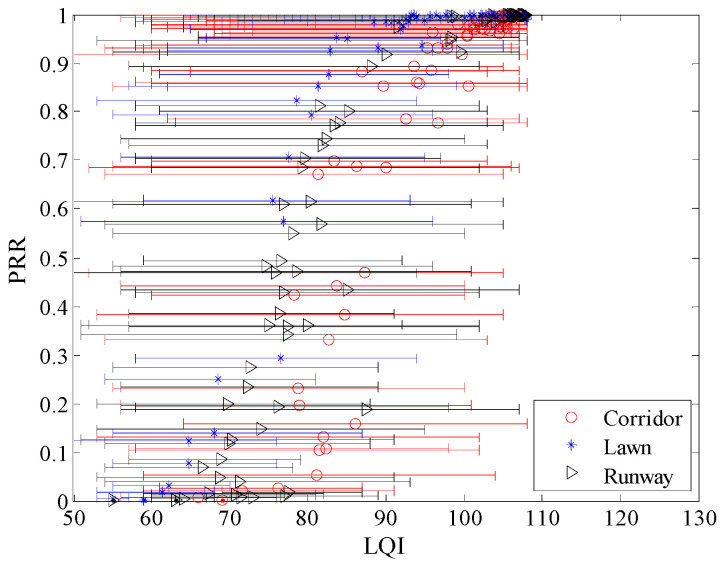
LQI vs. *PRR* in different environments.

**Figure 20:**
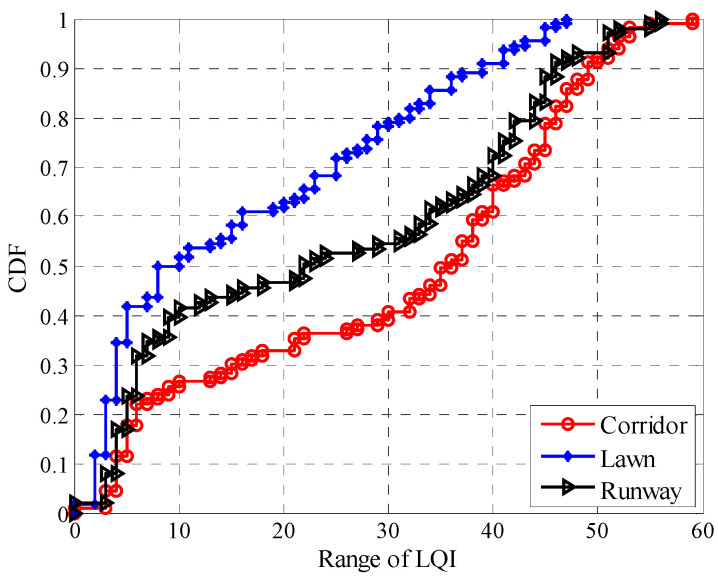
CDFs of the fluctuation range of LQI in different environments.

Figure 19 shows the relationship between LQI and *PRR* in different environments, including the minimum, maximum, and mean value of LQI. It can be seen that the trend of change between LQI and *PRR* is basically the same for the three different environments. In terms of *μ_lqi_*, *PRR* increases as *μ_lqi_* increases. To describe the impact of environmental changes on the fluctuation of LQI, the fluctuation ranges of LQI in different environments were statistically obtained, and their CDFs are shown in Figure 20. The fluctuation range of LQI is calculated by subtracting the minimum LQI from the maximum one. It can be seen that the maximum fluctuation ranges of LQI in the runway and corridor are close to 60, while the maximum fluctuation range of LQI in the artificial lawn is less than 50. The fluctuation of LQI in the corridor is the most violent, while the fluctuation in the artificial lawn is the gentlest. The fluctuation of LQI in the runway is between them. These relationships are consistent with the fluctuation of *RSSI* in the three environments. Considering the linear relationship between LQI and *SNR* [32], this consistency is not difficult to understand.

**Figure 21:**
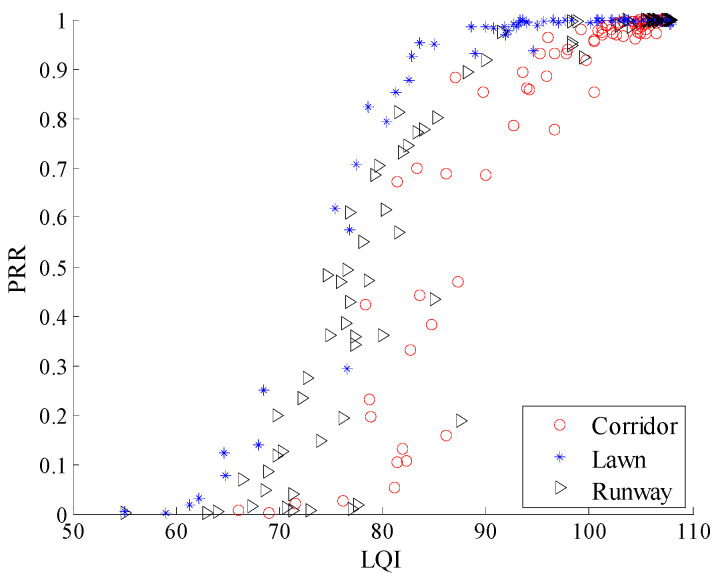
*μ_lqi_* vs. *PRR* in different environments.

To observe the environmental impact on the relationship between LQI and *PRR* more clearly, the relationship between *μ_lqi_* and *PRR* is shown in Figure 21. There already exist some studies which utilize the relationship between LQI and *PRR* to estimate link quality [6,15,16,17,18,26,27,28,30]. It can be seen that there is no obvious difference among the relationships between *μ_lqi_* and *PRR* in different environments. This means that the relationship between *μ_lqi_* and *PRR* is almost unaffected when the environment changes.

#### 5.4.2. Environmental Impacts on LQI Based LQEs

**Figure 22:**
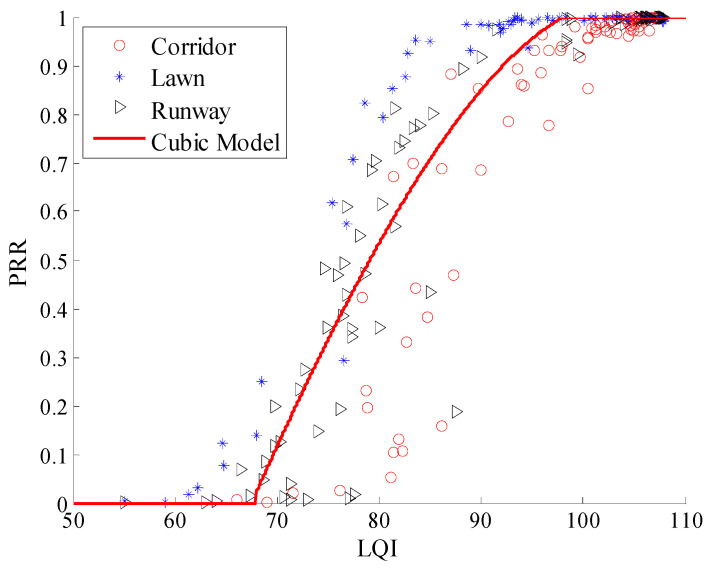
Effects of the cubic model in different environments.

**Figure 23:**
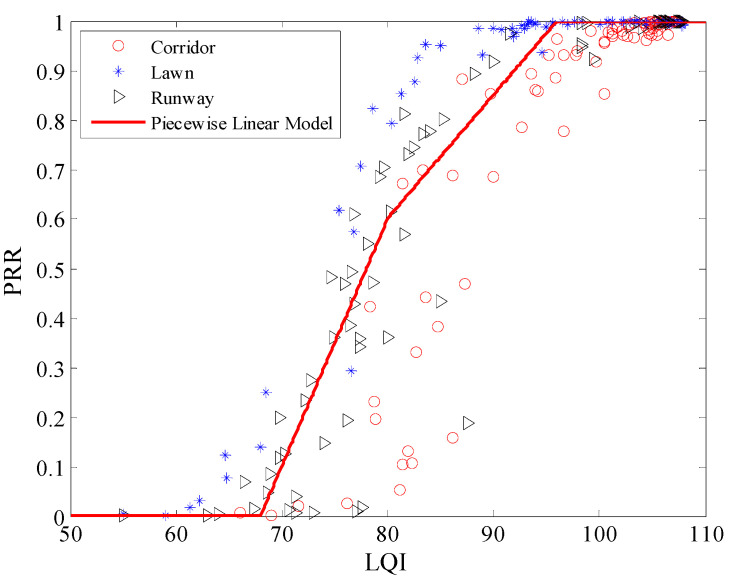
Effects of the piecewise linear model in different environments.

**Figure 24:**
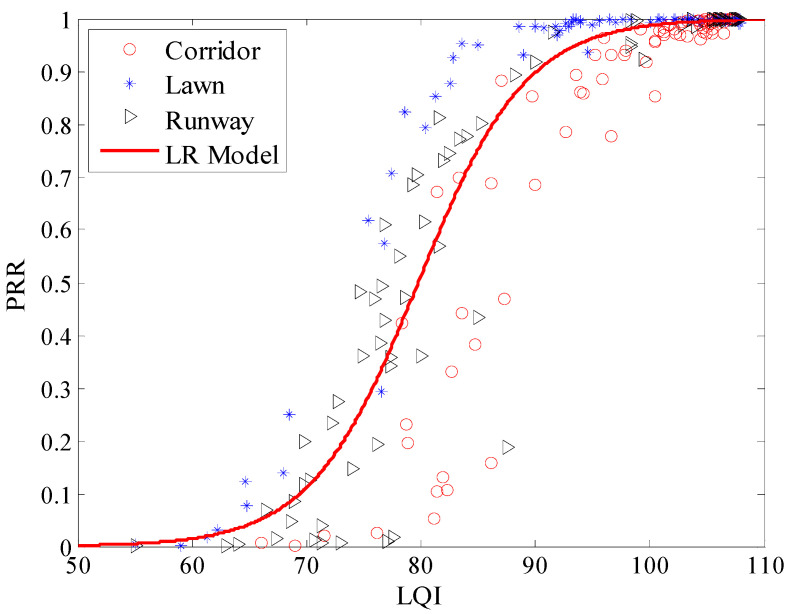
Effects of the LR model in different environments.

Using Equation (12), the Cubic model between *μ_lqi_* and *PRR* in three environments was plotted, as shown in Figure 22. It is clear that all the measured data from the three environments are basically coincident with the Cubic model. Using Equations (13) and (14), the piecewise linear model and LR model in the three environments were also plotted respectively, are shown in Figure 23 and Figure 24. It is clear that all the measured data from the three environments are also basically coincident with two models.

To describe the environmental impact on LQI based LQEs quantitatively, RMSE of the estimated *PRR* and real *PRR* in the three environments were calculated, as shown in Table 6. It can be seen that RMSEs of the three environments are almost the same, no matter whether the model is. Compared with that in the runway, RMSE of the Cubic model in the artificial lawn reduces by 19.63%, but increases by 20.94% in the corridor. RMSE of the piecewise linear model in the artificial lawn reduces by 28.30%, but increases by 30.12% in the corridor. RMSE of the LR model in the artificial lawn reduces by 22.14%, but increases by 23.00% in the corridor. That is to say, the mapping model between *μ_lqi_* and *PRR* constructed in a specific environment can be directly used in other environments.

### 5.5. Main Conclusions and Deep Analysis

According to the analysis in Section 5.1, Section 5.2, Section 5.3 and Section 5.4, environmental impacts on hardware-based LQEs in WSNs are summarized as follows:

- **Conclusion 1.** It is not accurate to estimate *PRR* using communication distance, and they may be useless when changing environments.
- **Conclusion 2.** When the environment changes, the fluctuation range of *RSSI* and *SNR* will be affected and that of LQI is almost unchanged.
- **Conclusion 3.***RSSI* based LQEs may degrade when the environment changes. Fortunately, this degradation is mainly caused by the change of background noise, which could be compensated conveniently.
- **Conclusion 4.** The best environmental adaptability is gained by LQI and *SNR* based LQEs, as they are almost unaffected when the environment changes.

**Figure 25:**
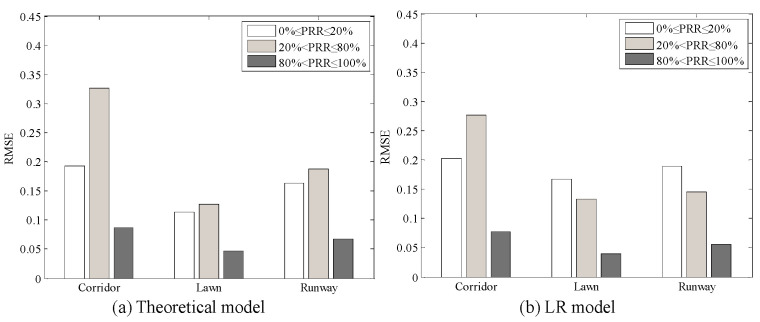
RMSEs of *SNR* based LQEs in different regions.

**Figure 26:**
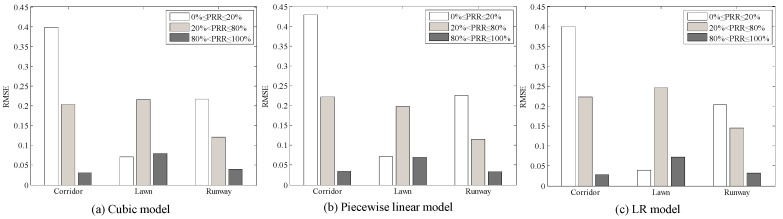
RMSEs of LQI based LQEs in different regions.

Combined with Figure 7, Figure 12 and Figure 19, it can be seen that the fluctuation range of *SNR* and *RSSI* is significantly smaller than that of LQI with the same *PRR*. This indicates that a larger window is needed to smooth LQI, which will inevitably affect the agility of LQI based LQEs. As can be seen from Figure 8, Figure 15 and Figure 21, when *PRR* changes from 20% to 80%, *μ_lqi_* corresponds to a range from about 70 to 100, while *μ_snr_* and *μ_rssi_* correspond to a range from about 1.8 dB to 4.5 dB and from about −95 dBm to −91 dBm, respectively. This indicates that using *μ_lqi_* to estimate *PRR* in the transitional region has a better resolution than *μ_snr_* and *μ_rssi_*, which means higher accuracy in the transitional region. To verify this conclusion, RMSEs in the connected region, transitional region, and disconnected region for *SNR* and LQI based LQEs were plotted, as shown in Figure 25 and Figure 26, respectively. It is shown that LQI based LQEs are really more accurate in the transitional region than *SNR* based LQEs, especially for the environments with higher fluctuation ranges of LQI and *SNR*. Therefore, we have one more conclusion:

- **Conclusion 5.** LQI based LQEs are more accurate than *SNR* based ones in the transitional region. Nevertheless, compared with *SNR*, the fluctuation range of LQI is much larger, which needs a larger smoothing window to converge. In addition, the calculation of LQI is typically vendor-specific [2]. Therefore, the tradeoff between accuracy, agility, and convenience should be considered in practice.

The superiority of LQI over the remaining indicators can be explained as follows: For the radio chip we used, LQI and *RSSI* are both calculated over the first eight symbols of the incoming packet, following the start of the frame delimiter [13]. *RSSI* represents the average radio signal power received during these eight symbols, and it includes both the useful signal and eventual interference. The key drawback of *RSSI* is that it is a measure of raw electromagnetic energy on the channel, which does not distinguish the useful signal from interference and does not consider the signal correctness. That’s why *SNR* based LQEs are more accurate than *RSSI* based ones because *SNR* considers the influence of noise and interference to some extent. On the other hand, each of the eight symbols is correlated with all 16 possible chip sequences, and the closest match is chosen for decoding. Then, LQI is calculated by the chip error rate with respect to the closest match [33]. In other words, LQI reflects the signal quality, not just the signal strength. Therefore, it has a better and more stable correlation with *PRR*. Therefore, LQI based LQEs are more susceptible to changing environments than *RSSI* and *SNR* based ones.

## 6. Conclusions and Future Works

To satisfy the performance requirements of WSNs, agile, and accurate link quality estimation is necessary. To achieve this goal, hardware-based LQEs are usually employed which depend on mapping models between *PRR* and some indirect metrics such as the *SNR*, *RSSI*, and LQI. However, existing studies did not consider the impacts of environmental changes on the applicability of these estimators. To solve this problem, three different environments are chosen in this paper, and environmental impacts on typical hardware-based LQEs are analyzed quantitatively.

The experimental results expose that the traditional calculation method of *SNR* used in existing studies is problematic. The transceiver only measures the power at the antenna without attempting to distinguish whether it is due to signal or noise. Therefore, the noise power also contributes to the *RSSI* value, which makes the actual *SNR* is nonlinear with *RSSI* and noise power, especially when *SNR* is small. For this problem, a more reasonable calculation method is proposed. It is shown that after correction, the *SNR* based theoretical model is more accurate, which makes the LQEs based on this model much more valuable.

It is not accurate to estimate *PRR* using communication distance and may be useless when the environment changes. The performance of *RSSI* based LQEs may degrade when the environment changes. Fortunately, it could be compensated conveniently by measuring the background noise or carrying out normalization to *RSSI*. The best environmental adaptability is gained by LQI and *SNR* based LQEs, as they are almost unaffected when the environment changes. Moreover, LQI based LQEs are more accurate than *SNR* based ones in the transitional region. On the other hand, when the environment changes, the fluctuation range of *RSSI* and *SNR* will be affected and that of LQI is almost unchanged. However, compared with *SNR* and *RSSI*, the fluctuation range of LQI is much larger, which needs a larger smoothing window to converge. In addition, the calculation of LQI is typically vendor-specific. Therefore, the tradeoff between accuracy, agility, and convenience should be considered in practice.

In the future, the impacts of node configurations (in other words, heterogeneous nodes) on LQEs will be explored to gain a deeper understanding, including the antenna height, frequency channel, transmit power, antenna angle, and others.

## Figures and Tables

**Table 1:** Main features of existing studies.

Ref.	RF Chip (Node Type)	Experimental Environment	Parameters	Modeling Method
[3]	N/A (Berkeley Mote)	indoor	*PRR*	N/A
[4]	CC2420 (TelosB)	outdoor (garden)	*SNR*, *PRR*	N/A
[5]	CC2420 (TelosB)	outdoor (500 kV substation), indoor (underground transformer vault and main power control room)	*SNR*, *PRR*, RNP	N/A
[6]	CC2550 (N/A)	indoor (industrial environment)	*SNR*, LQI, *PRR*	N/A
[7]	CC2420 (TelosB)	indoor (corridor), outdoor (rooftop and playground)	*PRR*	N/A
[8]	CC2420 (N/A)	indoor (office)	*SNR*, *PRR*	Pre-calibrated *SNR*-*PRR* relationship
[9]	RFM TR1000 (Mica)	indoor (office), outdoor (park and parking lot)	Distance, *PRR*	N/A
[10]	CC2420 (MicaZ, TelosB)	indoor (office), outdoor (dry lake)	*RSSI*, *PRR*	N/A
[11]	CC2420 (Imote2)	outdoor (road)	Distance, *PRR*	Fitting model
[12]	CC2530 (N/A)	outdoor (distribution substation, low-voltage transformer, and distribution lines in residential area)	Distances, *SNR*, *RSSI*, *PRR*	Theoretical model
[15]	CC1101 (N/A)	single environment	*RSSI*, LQI, *PRR*	N/A
[16]	CC2420 (TelosB)	outdoor	LQI, *PRR*	Cubic model
[17]	CC2420 (N/A)	indoor	LQI, *PRR*	Cubic model
[18]	CC2420 (TelosB)	indoor (laboratory)	LQI, *PRR*	Piecewise linear model
[19]	MRF24J40 (N/A)	indoor (industrial environment)	*RSSI*, *PRR*	Polynomial regression
[20]	CC2530 (N/A)	indoor, outdoor (square and grove)	*RSSI*, *PRR*	Logistic regression
[21]	CC2420 (MicaZ, TelosB)	indoor (office and a small home), outdoor (dry lake)	*SNR*, *RSSI*, LQI, *PRR*	N/A
[22]	CC2530 (N/A)	outdoor (smart grid)	*SNR*, *RSSI*, *PRR*	Theoretical model
[23]	CC2420 (TelosB)	indoor (office), outdoor (park, square)	*SNR*, *PRR*	Theoretical model
[24]	AT86RF231 (N/A)	outdoor (industrial)	*SNR*, *PRR*	Theoretical model
[25]	CC2420 (N/A)	indoor (corridor), outdoor (rooftop and playground)	*SNR*, LQI, *PRR*	Logistic regression
[26]	CC2420 (Tmote Sky)	indoor, outdoor	*SNR*, *RSSI*, LQI, *PRR*	Logistic regression
[27]	CC2420 (Tmote Sky)	indoor (office building)	*SNR*, *RSSI*, LQI, *PRR*	Logistic regression
[28]	N/A	different environments	LQI, *PRR*	N/A
[29]	CC2420 (TelosB)	indoor	*RSSI*	N/A
[30]	CC2420 (TelosB)	outdoor (road)	*RSSI*, LQI, *PRR*	N/A

**Table 2:** Relevant parameters of Equation (4).

Parameter	Value
*L_c_*	31.6899 dB
*n*	1.5871
*σ*	1.0081 dB
*P_n_*	−98.37 dBm

**Table 3:** The root mean square errors (RMSEs) of the mapping models in different environments.

	Runway	Lawn	Corridor
Theoretical model	0.2862	0.4507	0.5304
Fitting model	0.3191	0.4402	0.4474

**Table 4:** RMSEs of *RSSI* based link quality estimators (LQEs) in different environments.

	Runway	Artificial Lawn	Corridor
LR model	0.1161	0.1634	0.2693
PR model	0.1284	0.1455	0.1129

**Table 5:** RMSEs of *SNR* based LQEs in different environments.

	Runway	Artificial Lawn	Corridor
Theoretical model before calibration	0.3009	0.1909	0.1749
Theoretical model after calibration	0.1253	0.0609	0.1442
LR model after calibration	0.1154	0.0659	0.1284

**Table 6:** RMSEs of LQI based mapping model in different environments.

	Runway	Lawn	Corridor
Cubic model	0.1146	0.0921	0.1386
Piecewise linear model	0.1152	0.0826	0.1499
LR model	0.1152	0.0897	0.1417
